# Role of electrolyte abnormalities and unmeasured anions in the metabolic acid‐base abnormalities in dogs with parvoviral enteritis

**DOI:** 10.1111/jvim.15749

**Published:** 2020-03-04

**Authors:** Richard K. Burchell, Arnon Gal, Ryan Friedlein, Andrew L. Leisewitz

**Affiliations:** ^1^ Veterinary and Biomedical Sciences James Cook University Townsville Queensland Australia; ^2^ Department of Veterinary Clinical Medicine College of Veterinary Medicine, University of Illinois at Urbana‐Champaign Urbana Illinois USA; ^3^ Department of Companion Animal Clinical Studies, Faculty of Veterinary Science University of Pretoria Pretoria South Africa

**Keywords:** acid‐base, canine parvovirus, Henderson‐Hasselbalch, strong ion model

## Abstract

**Background:**

The strong ion model (SIM) is an alternative paradigm in the characterization of acid‐base disturbances particularly in complex disorders.

**Hypothesis/Objectives:**

To compare the acid‐base changes in dogs with parvoviral enteritis (PE) using the Henderson‐Hasselbalch (HH) approach, with 2 strong ion approaches.

**Animals:**

Forty‐four dogs with PE, and 16 age‐matched control dogs.

**Methods:**

Prospective controlled observational study. Acid‐base status was evaluated using the HH model, Fencl‐Stewart (FS) approach and a validated strong ion model (VDM). The acid‐base changes according to each model were classified and compared. Statistical correlations between pH, CO_2_, and various SIM variables were performed, as well as between the sum of effects (SOE) of the SIM and the individual variables comprising the SOE.

**Results:**

The HH model identified acid‐base disorders in 31/44 cases of which 16/31 were mixed with metabolic acidosis and concurrent respiratory alkalosis the most common (10/31). Using the FS approach, metabolic changes were present 36/42 cases, with changes in free water (FW), chloride, and unmeasured anions (UA) being the most prevalent. Both FW and UA correlated well with pH; however, UA were most consistently abnormal in severe acidemia. Similarly to the HH, the VDM detected acid‐base disturbances in 28/44 cases. Major contributors to the acid‐base changes were hyponatremia, hypochloremia, and A_tot_ acidosis because of elevated globulins and increased UA.

**Conclusions and Clinical Importance:**

Acid‐base changes are common and complex in dogs with PE, and were easier to understand using a SIM paradigm. Increases in UA have not been documented in PE in dogs.

**Abbreviations**
A_tot_: total concentration of nonvolatile weak acids
FS: Fencl‐Stewart
HH: Henderson‐Hasselbalch
PE: parvoviral enteritis
SID: strong ion difference
SIG: strong ion gap
SIM: strong ion model
SOE: sum of effects
UA: unmeasured anions
VDM: validated data method

## 1 INTRODUCTION

Acid‐base assessment is predominately used in the critical care setting, as a conceptual paradigm of critical illness severity.^1^, ^2^ Because acid‐base homeostasis is tightly controlled,^3^ acid‐base disease represents a physiological derangement that has overcome compensatory mechanisms and can be looked at as a critical care “window.” Resolution of the acid‐base disorder can thus be used to track the success of treatment.^1^ Parvoviral enteritis (PE) in dogs results in marked fluid and electrolyte losses,^4^, ^5^, ^6^ protein loss, and sepsis^7^ all of which can result in marked acid‐base perturbations. Notwithstanding the prevalence of PE, the pathogenesis of acid‐base disturbances of PE have not been well described. Three previous studies utilizing the Henderson‐Hasselbalch (HH) model yielded discordant results regarding the etiology of the acid‐base disease. One study reported lactate acidosis^8^ (with concomitant acidemia) as the predominate change, whereas in another study metabolic alkalosis was more commonly observed.^9^ A third study demonstrated mostly mixed acid‐base disorders with a neutral pH.^6^ In addition, in a retrospective study using the strong ion model (SIM), chloride and albumin were found to be important contributors to the metabolic acid‐base changes,^5^ but the retrospective nature of that study precluded the calculation of the anion gap (AG) and unmeasured anions (UA), and the authors were also unable to compare these findings to the HH model. The authors of that study concluded that the acid‐base changes of PE were complex and multifactorial and conjectured that they would be incompletely explained by the HH model.^5^ The HH model summarizes the metabolic compartment in the HC$O_{3}^{-}$ deflection from normal, which might be influenced by: metabolic acidosis/alkalosis or by the reciprocal compensation to primary changes in pCO_2_. Therefore, mixed disorders might be concealed within a modest deviation of HC$O_{3}^{-}$ that belies the true severity of a metabolic disorder. A paucity of data exists regarding the role of UA in veterinary medicine. UA are associated with a lethargic demeanor in calves,^10^ but studies in dogs are scarce. In humans there is a relationship between an increased strong ion gap (SIG) and elevated inflammatory cytokines, 11 but this has not been shown in veterinary patients. Increased UA correlates with death in humans 12, 13, 14 but in 1 retrospective study in dogs none of the acid‐base perturbations, including elevated UA, were considered to confer a prognostic advantage. 15 The purpose of the present study was to describe the acid‐base changes in PE using both the HH and SIM models, and to compare and contrast the findings.

## 2 MATERIALS AND METHODS

### 2.1 Animals and study design

The study was conducted at the Onderstepoort Veterinary Academic Hospital, University of Pretoria and was approved by the Animal Ethics and Care Committee (approval number V068‐14). Puppies with PE were prospectively recruited into the study and samples were collected on the day of admission before the administration of any treatment. The diagnosis of PE was confirmed with a point‐of‐care sandwich ELISA (SNAP Parvo 2015, Idexx Laboratories, One Idexx Drive, Westbrook, Maine) in puppies that were unvaccinated and under 6 months of age. Venous blood (1 mL) was collected in heparinized blood gas syringes (BD A‐Line, arterial blood collection syringe, Becton, Dickinson and Company, UK) and blood gas was performed immediately by 2 of the coauthors (AL and RF) within 15 minutes of collection on a bench‐top blood gas analyzer (Rapidpoint 405 ‐ Bloodgas, Siemens, PO Box 198 Isando 1600, South Africa). A further 2 to 3 mL of blood was collected in plain serum tubes and submitted for serum biochemistry. These data were generated by the University of Pretoria, Department of Veterinary Clinical Pathology (Cobas Integra 400 Plus Chemistry, Roche, PO Box 1927 Randburg 2125, South Africa). To generate acid‐base values for comparison, 16 healthy, age‐matched control dogs were used, which consisted of client‐owned healthy dogs presented for vaccination.

### 2.2 Henderson‐Hasselbalch analysis

Apparently healthy dogs were used to generate data for comparison for the blood gas and acid‐base variables, and were calculated using the mean ± 2 standard deviations; these data are shown in Table 1. This approach was based on the methodology used in a previous study.^16^ These data were used to compare the changes in the blood‐gas and acid‐base variables, as well as the calculated acid‐base changes to the PE group and served as the normal reference ranges (Table 2).

**Table 1:** Blood gas and electrolyte data of PE affected and control dogs

	PE dogs (n = 44) (mean ± SD)	Control dogs (n = 16) (mean ± SD)	*P* value
Measured variables			
Total protein (g/L)	50.0 ± 6	45.0 ± 4	.002*
Albumin (g/L)	28.5 ± 5.0	29.8 ± 4.5	.28
Globulin (g/L)	20.9 ± 6.7	15.4 ± 2.4	<.001*
Sodium (mmol/L)	139.5 ± 5.6	143 ± 1.8	.004*
Potassium (mmol/L)	3.8 ± 0.5	4.5 ± 0.4	<.001*
Ionized calcium_actual_ (mmol/L)	1.32 ± 0.07	1.37 ± 0.06	.01*
Chloride (mmol/L)	102.9 ± 6.6	108.7 ± 2.2	.001*
Phosphate (mmol/L)	2.5 ± 0.61	2.9 ± 0.39	.003*
Lactate (mmol/L)	1.6 ± 0.7	1.7 ± 0.8	.92
pH	7.34 ± 0.07	7.35 ± 0.04	.81
pCO_2_ (mm Hg)	42.4 ± 3.1	44.6 ± 4.2	.47
Calculated variables			
HC$O_{3}^{-}$ (mmol/L)	23.5 ± 3.7	23.3 ± 2.3	.92
Base excess (mEq/L)	−2.4 ± 3.6	−1.94 ± 2.4	.31
Base excess_corrected_ (mEql/L)	−0.37 ± 3.9	−1.0 ± 1.9	.70
Anion gap (mEq/L)	16.9 ± 3.1	15.9 ± 3.1	.35
Fencl‐Stewart approach			
Free water effect (mEq/L)	−1.1 ± 1.4	−0.14 ± 0.5	.005*
Corrected chloride (mEq/L)	106 ± 3	109.3 ± 2.7	.004*
Chloride effect (mEq/L)	2.7 ± 4.1	−0.1 ± 2.0	.004*
Phosphate effect (mEq/L)	0.52 ± 1.2	−0.13 ± 0.7	.003*
Albumin effect (mEq/L)	0.20 ± 1.9	−0.26 ± 1.6	.28
Lactate effect (mEq/L)	−1.6 ± 0.7	−1.7 ± 0.8	.91
Unmeasured anions (mEq/L)	−3.1 ± 3	0.7 ± 2	.001*
Unmeasured anions BE_corrected_ (mEq/L)	−1.78 ± 3.25	1.16 ± 1.8	.004*
Sum of effects (mEq/L)	1.1 ± 3.5	2.4 ± 2	.001*
Validated data method			
SID3 (mEq/L)	41 ± 3	39 ± 2	.2
SID4 (mEq/L)	39 ± 3	41 ± 2	.06
A_tot‐albumin_ (mEq/L)	11.9 ± 2.1	12.5 ± 1.9	.29
A_tot‐total protein_ (mEq/L)	12.6 ± 1.5	11.3 ± 1.1	.002*
SIG_alb_ (mEq/L)	0.15 ± 6.1	0.81 ± 2.1	.2
SIG_alb‐simplified_ (mEq/L)	−1.8 ± 5.7	−1.17 ± 1.8	.12
SIG_TP_ (mEq/L)	0.75 ± 5.4	−0.84 ± 2.4	.24
SIG_TP‐simplified_ (mEq/L)	−1.2 ± 5.4	−2.5 ± 2.2	.32

*Note: P* values marked with an asterisk are statistically significant.

**Table 2:** Blood‐gas and calculated acid‐base variables for diagnostic comparison based on data from 16 age matched puppies

Variable	Values
pH	7.33‐7.42
pCO_2_ (mmHg)	38‐47
HC$O_{3}^{-}$ (mmol/L)	19‐28
Base excess (mEq/L)	−5.1 to 2
Base excess_corrected_ (mEql/L)	−3.7 to 3.6
Lactate (mmol/L)	<−2.9
Calculated SIM variables	
Anion gap (mmol/L)	10‐21
SIG	−5.4 to 2.3
Chloride effect	−3.3 to 3.5
Free water effect	−0.8 to 0.5
Phosphate effect	−1.5 to 0.8
Albumin effect	−2.4 to 2.4
Lactate effect	<−2.9
Unmeasured anions	<−4.3
SID_3_	36‐43
SID_4_	37‐42
A_tot‐albumin_	9‐16
A_tot‐total protein_	9.5‐13
SIG_alb_	−2.9 to 4.3
SIG_alb‐simplified_	−4 to 2.5
SIG_TP_	−4 to 4
SIG_TP‐simplified_	−5 to 2.2

An HH acid‐base appraisal was conducted, based on assessment of the pH, pCO_2_, and HC$O_{3}^{-}$, using the data generated from the control dog group. In each case the corresponding change in pCO_2_ or HC$O_{3}^{-}$ was evaluated and compared with expected compensation values.^16^ When these values were outside of expected compensation range (±2 mmHg or mmol/L, respectively), or when changes in the respiratory and/or metabolic compartment were observed in the face of a normal pH, a mixed disorder was diagnosed.^17^ Furthermore, the AG was calculated in each case and when the AG exceeded the top end of the reference range, an increased AG acid‐base disorder was diagnosed. A final acid‐base diagnosis according to the HH model could therefore be reached based on whether the primary change was respiratory/metabolic/mixed. The HH outcomes were visually displayed as Venn diagrams.

### 2.3 Strong ion model

#### 2.3.1 Fencl‐Stewart approach

The data were assessed using a simplified approach^16^ based on the original work of Fencl and Leith.^18^ The authors used this model because in our experience it is 1 of the most popular approaches used by our colleagues. However, this model has not been validated in dogs. It has been shown previously that there are key differences in acid‐base balance variables between dogs and humans, particularly with respect to the buffering properties of proteins^19^ and between the nomograms from which the base excess is derived^20^ (which is used to calculate UA). The comparator data used to generate the calculated acid‐base values for the AG and SIM data in the control dog group were derived from the mid‐normal reference values of the laboratory of the Department of Veterinary Clinical Pathology at the University of Pretoria. The effects of chloride, free water (sodium), albumin, phosphate, and lactate were calculated and summated (Table 3). The sum of effects (SOE) was subtracted from the base excess (BE) in order to determine the presence of UA. Each of the effects was correlated with pH and the SOE to assess the relationship of each metabolic component with the overall acid‐base changes. The number of dogs with changes in each of the aforementioned categories was quantified. To reach a final diagnosis, the chloride, sodium, and lactate effect were evaluated for changes in SID (alkalosis/acidosis), and the albumin and phosphate effect were summated to yield the A_tot_. The acid‐base changes according to the SIM were visually depicted as Venn diagrams. Lastly, to account for possible species differences in the BE (which could affect UA) the blood gas machine calculated BE was compared to the BE_corrected_ that was calculated using the Van Slyke^21^ equation (BE = 0.9287(HC$O_{3}^{-}$ ‐ 24.4 mmol/L + 14.83 [pH ‐7.4]) using a pH 7.35 and a HC$O_{3}^{-}$ of 23 mmol/L (based on our control dog data) as normal values (as opposed to 7.4 and 24.4 mmol/L). For the purposes of diagnosis the blood gas machine (Rapidpoint 405) derived BE values were used to calculate UA.

**Table 3:** Formulas for calculated variables

Model	Formula
Anion gap	([Na^+^] + K^+^]) ‐ ([HC$O_{3}^{-}$] + [Cl^−^])
Fencl‐Stewart approach	
Albumin effect	3.7 × ([alb]_mid‐normal_ − [alb]_patient_)
Chloride effect	[Cl^−^]_mid‐normal_ − [Cl^−^]_corrected_
Corrected chloride	[Cl^−^]_measured_ × ([Na^+^]_mid‐normal_/[Na^+^]_patient_)
Free water effect	0.25([Na^+^] _patient_ − [Na^+^]_mid‐normal_)
Lactate effect	−1 × [lactate]
Phosphate effect	0.58([phosphate]_mid‐normal_ − [phosphate]_patient_)
Sum of effects	Free water effect + chloride effect + phosphate effect + albumin effect + lactate effect
Unmeasured anions	Base excess − sum of effects
Validated data model	
SID_3_	([Na^+^] + K^+^]) − [Cl^−^]
SID_4_	([Na^+^] + K^+^]) − ([Cl^−^] + [L‐lactate])
A_tot‐albumin_	[albumin] × 0.42 mEq/g
A_tot‐total protein_	[total protein] × 0.25 mEq/g
SIG_alb_	[albumin] × (0.348 × 0.469/20) − anion gap
SIG_alb‐simplified_	[albumin] × 0.49 − anion gap
SIG_TP_	[total protein] × (0.206 × 0.273/{1 + 10^[7.7‐pH]^}) − anion gap
SIG_TP‐simplified_	[total protein] × 0.29 − anion gap

#### 2.3.2 Use of a validated data model

As mentioned earlier, the Fencl‐Stewart (FS) approach has not, to the authors’ knowledge, been validated for use in dogs, and is premised on data derived from work in humans. Accordingly, the authors employed a model using experimentally validated data.^19^ Briefly, the strong ion difference was calculated as shown in Table 3, and the A_tot_ was calculated using both albumin and total protein for comparison, because of the fact that globulins have been shown to contribute to the A_tot_ to a greater extent than is seen in humans.^19^ Lastly, the SIG was calculated to detect the presence of UA for comparison, with the UA as determined by the FS approach. Four calculations for SIG were used for comparison, based on equations developed for use in canine plasma.^19^ It has been previously shown that there are significant differences between SIG when it is calculated using different equations.^22^ The data generated in the PE group were compared to the control dog data to arrive at an acid‐base diagnosis (Table 2). Using this model the following acid‐base diagnoses could be reached based on whether SID, A_tot_, and SIG were increased/decreased according to Table 3; SID acidosis/alkalosis, A_tot_ acidosis/alkalosis, and the presence of UA acidosis as evidenced by an increase in the SIG. For the purposes of diagnostic comparison the SIG calculated from total protein was used, which factors a correction for pH into the equation.^19^

Both equations for SID (SID3 and SID4) were used to diagnose changes in SID. If 1 of the SID equations were abnormal a SID disorder was diagnosed. These data were displayed using Venn diagrams with the SIG representing the SIG calculated using total protein with a correction for pH. Furthermore, the delta chloride (Δchloride) and sodium (Δsodium) were calculated, because of the fact that we suspected that concurrent changes in sodium and chloride would be present, which would potentially neutralize the SID_3_ and SID_4_. This was calculated by subtracting the PE dogs’ serum sodium and chloride from the normal value derived from our control population (Table 2). Changes greater than 2 SDs from the median were considered clinically important.

### 2.4 Statistical analysis

The data were analyzed using a commercial statistics package (MedCalc Statistical Software version 18.10.2, MedCalc Software bvba, Ostend, Belgium). D'Agostino test for normality was performed. Data that were normally distributed were reported as mean ± SD. However, comparisons between the PE and control group were performed using the Mann‐Whitney *U* test, because of the small size of the control dog group. All correlations between the variables were performed using Spearman's rank correlation because of that variables such as the SIG has been shown to be nonlinearly associated with pH.^23^

## 3 RESULTS

Acid‐base and biochemical data from both the PE and control group, in addition to the calculated variables are shown in Table 1. There were no significant differences between the PE and control groups for any of the Henderson‐Hasselbalch (HH) variables. Significant differences were present for total protein (but not albumin), chloride, sodium, phosphate, ionized calcium, and potassium (*P* values shown in Table 1). Furthermore, there were a number of significant differences between the PE and control group in the strong ion approach variables, including chloride, free water, phosphate, the SOE, and UA. There were no significant differences between the BE and the BE_corrected_ for the control group (*P* = .63), but there was a significant difference between BE and BE_corrected_ for PE group (*P =* .01). There were also no significant differences between the UA using BE_corrected_ for the control group (*P* = .30) but there were for the PE group (*P* = .04).

### 3.1 Henderson‐Hasselbalch approach

**Figure 1:**
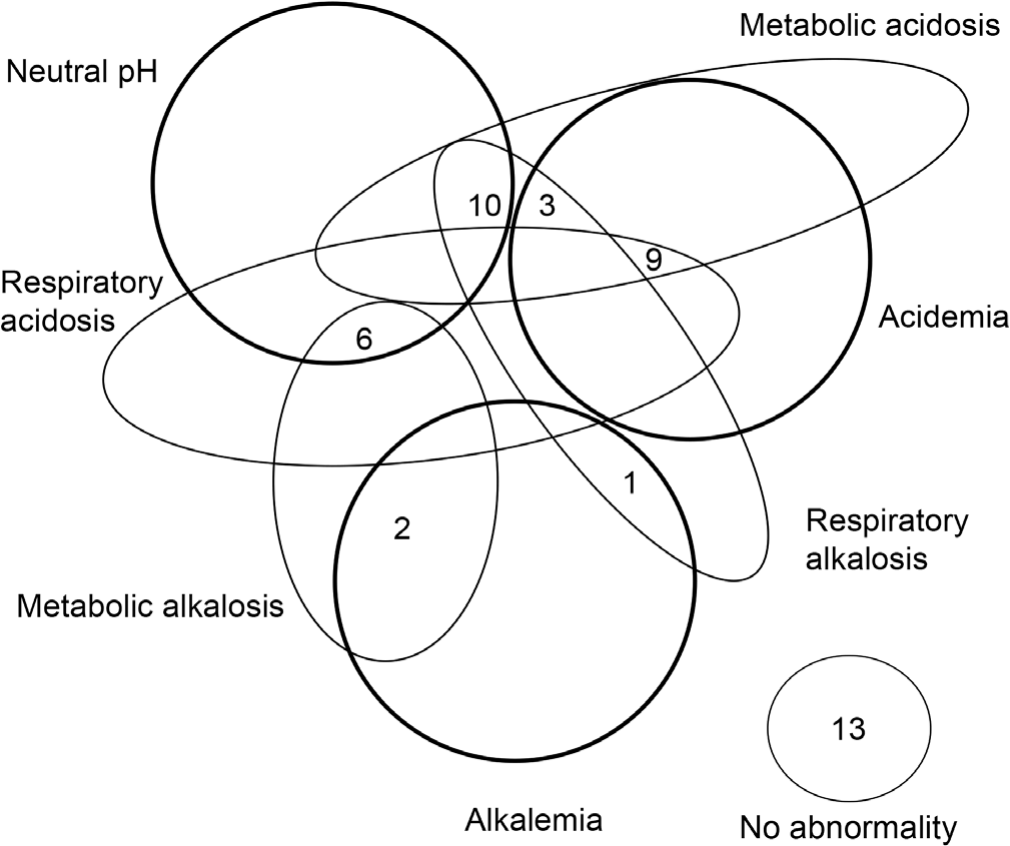
Venn diagram pictorial representation of the acid‐base changes in 44 puppies with PE according to the HH approach. The 3 darker circles represent pH neutral disorders, acidemia, and alkalemia. The oval circles denote the acid‐base processes and the intersections indicate concurrent processes are present. The numbers correspond to the number of cases with each process occurring. HH, Henderson‐Hasselbalch

The acid‐base disturbances according to the HH are shown in Figure 1. The most common pH change was acidemia (pH < 7.33) which occurred in 12/44 dogs. Only 3/44 dogs had an alkalemic pH, with the rest (29/44) having normal blood pH. Within the neutral pH group, 13/29 did not have an acid‐base abnormality according to HH. Of the remaining neutral pH dogs, mixed disorders were present, the most common of which was metabolic acidosis with concurrent respiratory alkalosis in 10/16 cases. Within the acidemic group, 7 were considered severely acidemic (pH < 7.3). The most consistent abnormality in the severely acidemic group was hypercapnia (6/7 cases). Blood pH and CO_2_ were strongly correlated (*P* = .001, rho 0.46). Interestingly, HC$O_{3}^{-}$ was only abnormal in 10 cases, with 5 of each metabolic acidosis and alkalosis, respectively. However, HC$O_{3}^{-}$ was outside of the expected compensation range in 9/12 cases that were acidemic, indicating a mixed disorder with a concurrent metabolic component. Mixed disorders were more frequently diagnosed than simple disorders (28/31 and 3/31). None of the dogs with acid‐base disorders had AGs outside of the control dog derived reference range.

### 3.2 Fencl Stewart approach

**Figure 2:**
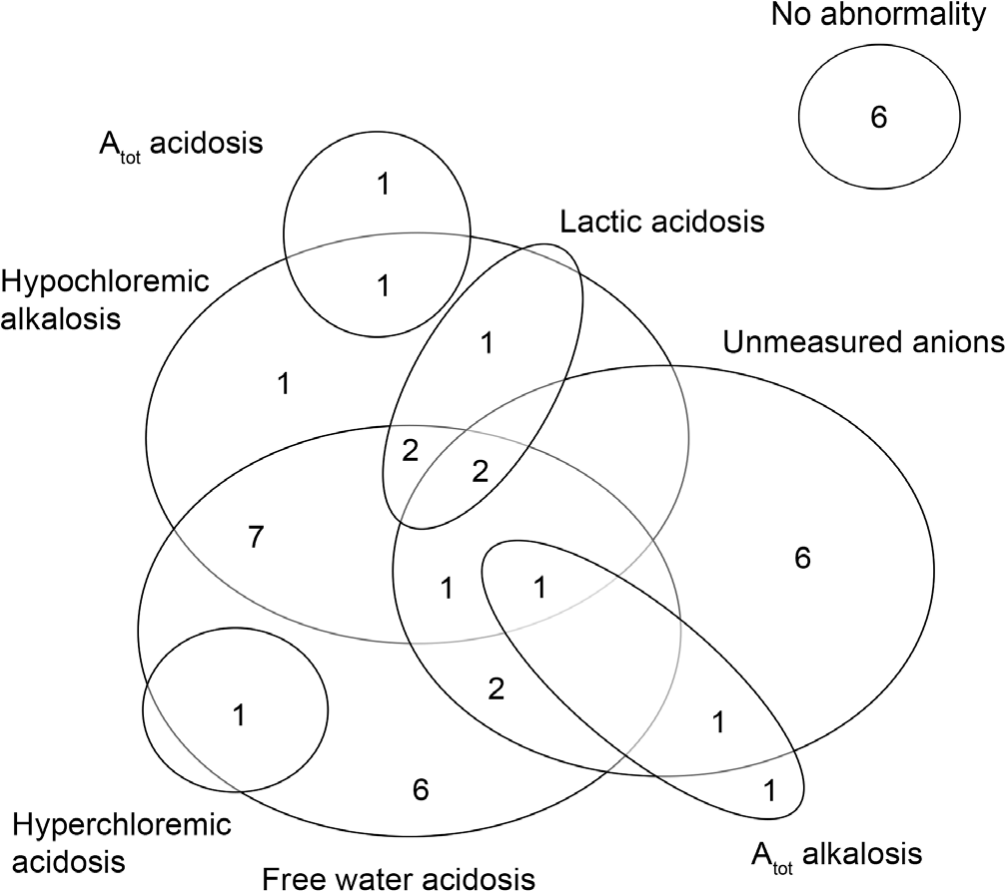
Venn diagram of 42 puppies with PE showing the relationship of the free water, chloride, lactate, A_tot_ in the metabolic acid‐base changes. Each of the circles represents a strong ion process, and the intersections between each show what concurrent acid‐base changes are present. The numbers in each of the circles represents the number of cases with each process occurring. A_tot_, total nonvolatile organic acids; FS, Fencl‐Stewart

Of the 44 dogs in the PE group, 42 had complete data sets to permit a FS analysis. Fewer cases were judged to have no acid‐base disease according to this approach (6/42) than according the HH (13/44). Of the 42 dogs with metabolic acid‐base abnormalities, complex metabolic disorders, involving perturbations in more than 1 of the metabolic compartments, were present in 28/36 cases (Figure 2).

**Figure 3:**
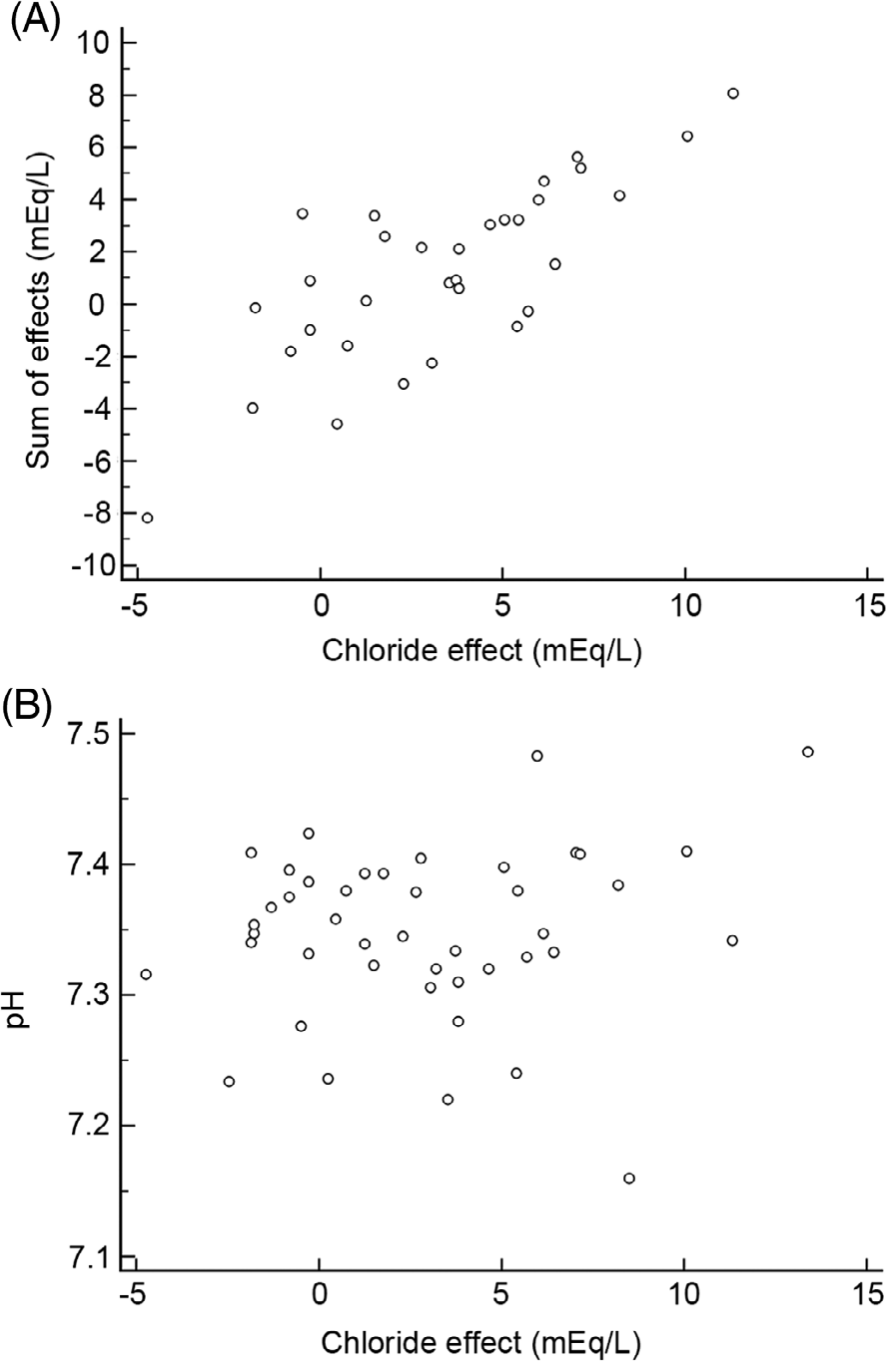
Spearman's rank correlation between chloride to SOE and pH. A, Correlation between chloride and SOE (*P* < .001; rho 0.79). B, Correlation between chloride and pH (*P* = .42; rho 0.12)

**Figure 4:**
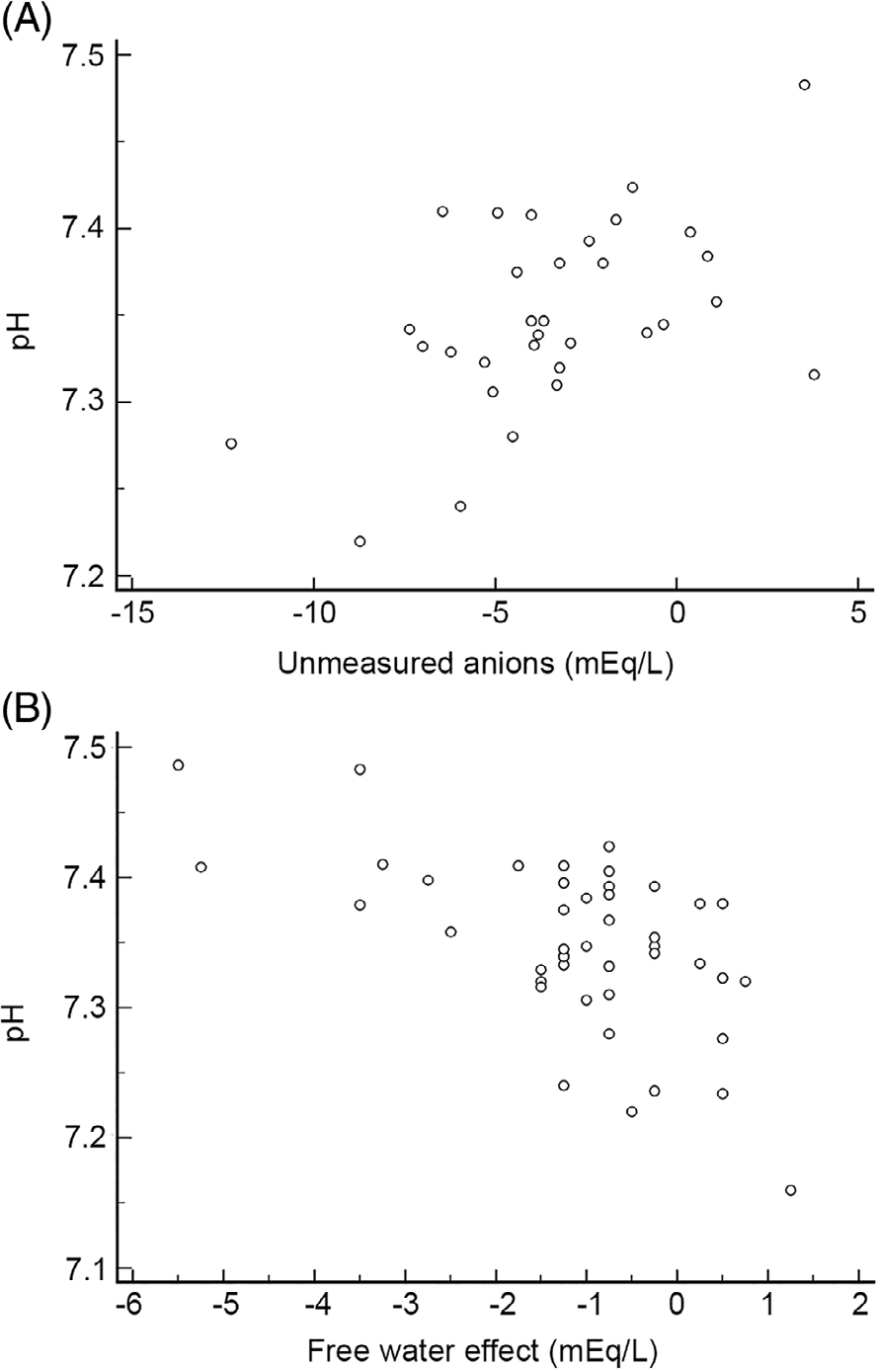
Spearman's rank correlation between the pH to unmeasured anions and the free water effect. A, Correlation between pH and unmeasured anions (*P* = .03; rho 0.0.4). B, Correlation between pH and the free water effect (*P* = .002; rho −0.46 respectively)

In the severely acidemic group 6/7 dogs had increased anions (≥ 4.3 mmol/L) and 5/6 were hypercapnic. Increases in UA were detected in 11 cases using this method. When the UA were calculated using the BE_corrected_ (pH 7.35, HC$O_{3}^{-}$ 23 mmol/L) UA were detected in 12 cases in total and in 6/7 of the severely acidemic dogs. Chloride was the most important determiner of the SOE (Figure 3) (*P* < .001; rho 0.79) but was not significantly correlated with blood pH (*P* = .42). Conversely, UA and free water were significantly correlated with blood pH (Figure 4) (*P* = .03; rho 0.4 and *P* < .001; rho −0.46 respectively). UA using BE_corrected_ was also significantly correlated with pH (*P* = .01). Changes in free water (sodium) were not significantly associated with the SOE. In addition, a free water acidosis was only present 1/7 severely acidemic cases. The SOE was also significantly associated with HC$O_{3}^{-}$ (*P* = .002, rho 0.61), and with blood pH (*P =* .03; rho 0.39). Blood lactate was not elevated in any of the severely affected dogs, and did not significantly correlate with the SOE or the blood pH.

### 3.3 Validated data method

**Figure 5:**
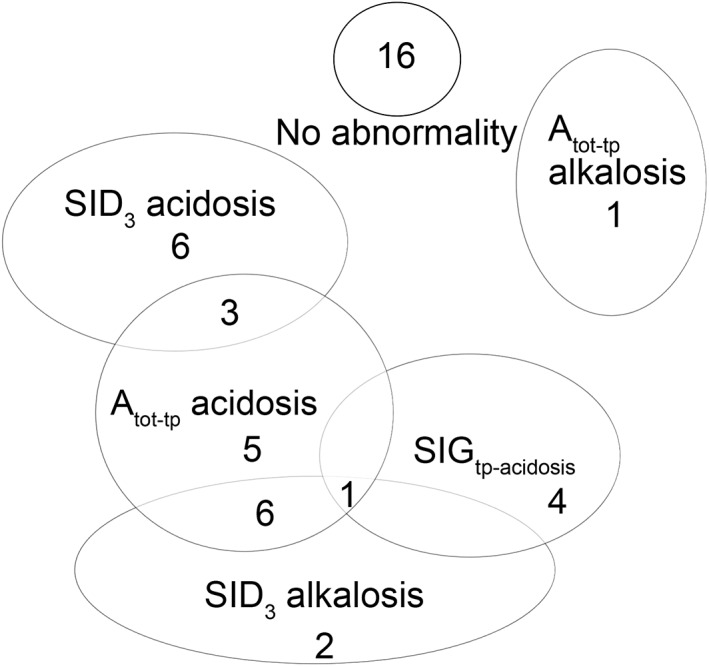
Venn diagram of 44 puppies with PE showing the relationship of changes in SID, A_tot_, and unmeasured anions (SIG) in the metabolic acid‐base changed. For the purposes of diagnostic comparison, the SIG equation derived from total protein was used, with a correction factor for pH (Table 2). Each of the circles represents a strong ion process, and the intersections between each show what concurrent acid‐base changes are present. The numbers in each of the circles represents the number of cases with each process occurring. A_tot_, total nonvolatile organic acids; SIG, strong ion gap; VDM, validated data model

Similar to the HH method, 16/44 cases were judged to have no acid‐base abnormality when the VDM was used. Perturbations of the SID were present in 18 cases with 9/18 cases of SID acidosis and 9/18 cases of SID alkalosis (Figure 5). Eight cases of SID acidosis were because of hyponatremia alone, and 1/9 was because of hyperchloremia. Within the SID alkalosis group, 1/9 were because of hypochloremia alone and in the remaining 8 cases, a hypochloremia and concurrent hypernatremia was present in 6/8 cases and hypernatremia alone, was present in 2/8 cases. Interestingly, in 7/13 cases that had no acid‐base change according to the VDM, the Δchloride and Δsodium were deemed to be abnormal but the eventual change in SID was within the reference range. All of these 7/13 cases were judged to have acid‐base abnormalities according to the FS approach. Furthermore, Δchloride and Δsodium identified a total of 18 cases of hypochloremia and 19 cases of hyponatremia. Moreover, both Δchloride and Δsodium were statistically significantly related to pH (*P =* .04, rho 0.33 and *P* = .002, rho 0.46 respectively).

Using the 4 equations for SIG developed for dogs by Constable and Stampfli,^19^ increases in unmeasured anions were present in 11, 5, 13, and 7/44 cases for the SIG_alb,_ SIG_TP_, SIG_albsimplified_, and SIG_TPsimplified_ respectively. All of the 5 cases identified with increases in UA by the SIG_TP_ method also had increases according to the FS method and according to the other 3 equations for SIG from the VDM. Of the 11 cases identified with UA by the FS method, only 1/11 did not have elevated UA according to at least 1 of the SIG equations from the VDM.

When the A_tot_ was estimated from the total protein, the most common abnormality was an A_tot_ acidosis (Figure 5), presumably because of a mild increase in globulins as evidenced by a significant difference for globulins between the PE and control group (*P <* .001).

## 4 DISCUSSION

These results reaffirm the conclusions of a previous retrospective study,^5^ which described complex mixed disorders according to the SIM, and postulated that these mixed disorders would be poorly explained according to the HH model. Clinicians remain divided as to the advantage of 1 acid‐base paradigm over the other, and thus the great trans‐Atlantic acid‐base debate^24^, ^25^ remains unresolved. Some researchers have suggested that the approaches are essentially the same, but are premised on different epistemological principles.^26^, ^27^ Many clinicians find acid‐base physiology an arcane field with no clear consensus as to how it should be interpreted and which model should be used. Moreover, acid‐base physiology is still conjectural, based largely on mathematical models, predicated on differing physicochemical hypotheses.^26^

However, assuming the veracity of the SIM paradigm we demonstrated a clear advantage of the SIM over HH in explaining complex mixed metabolic acid‐base perturbations in PE. We specifically chose PE as a disease model, due the diversity of expected perturbations to acid‐base homeostasis, which we suspected would have concomitant acidifying and alkalinizing effects, given the fact that diarrhea is often associated with acidemia,^10^, ^28^ and vomiting with alkalemia.^29^, ^30^ Based on previous work^5^ we anticipated changes in SID and A_tot_ would be present, but we did not anticipate the presence of UA in severe acidemia as was documented in this study. In accordance with a previous study, using the FS, chloride was the most strongly associated with the SOE (*P* < .001; rho 0.79) but it was not significantly correlated with blood pH (Figure 3). These results infer the importance of chloride in the changes of the metabolic compartment according to the FS approach, but the lack of association with pH suggested that it was part of a complex and mixed process. Conversely, UA and free water were significantly correlated with blood pH (*P* = 0.03; rho 0.4 and *P* = .002; rho: −0.46 respectively). Interestingly, hyponatremia has been associated with acidemia in calves with diarrhea^23^ but not in dogs. In this study, free‐water acidosis was present in 22/42 cases (Figure 2). However, in 13/22 cases a concurrent hypochloremic alkalosis was also present, which would have a neutralizing effect on blood pH.

The findings according to the VDM were similar to those of the FS approach, with the exception of A_tot_ changes. The VDM detected fewer cases with acid‐base abnormities using the SID, A_tot‐tp_, and SIG_tp_ alone. However, when the individual changes in chloride and sodium were scrutinized within the normal SID group, we were able to show that a concurrent hypochloremia (presumably from vomiting) and hyponatremia (presumably from diarrhea) tended to offset changes in the SID as determined by the SID_3_ and SID_4_. When the Δchloride and Δsodium were evaluated in dogs with normal SID (as calculated by the VDM), we were able to identify all of the cases that were judged to be abnormal according to the FS approach. We propose that when the VDM is used, clinicians are cognizant of neutralizing SID effects, and pay attention to the individual contributors of the SID when calculating this. We also propose that the VDM's diagnostic utility could be augmented by calculating the Δsodium and Δchloride in cases where concurrent changes might be present such as in PE. When UA were calculated using the FS approach and VDM equations, 11 cases were identified by the FS approach and the SIG_alb_. The SIG_alb‐simplified_ identified 13 cases with increases in UA, and the SIG_tp_ identified the fewest, with 5 cases judged to have increased UA. These results confirm the findings and conclusions of a previous study in healthy dogs^20^ that showed significant differences between SIG calculated using different equations, and emphasize the importance of using equations that have been validated for use in dogs. Further studies are necessary to determine which of these equations for SIG is the most appropriate to use in dogs in various disease processes. The fact that the VDM showed that an A_tot_ acidosis was the most common change further emphasized the need to use a standardized approach with species‐specific data validation, as these were likely underestimated by the FS approach which is derived from human data. According to the HH model, metabolic changes were common, which would be expected given the severe biochemical insults associated with vomiting, diarrhea, and sepsis. Interestingly, in many of these cases, the bicarbonate was inappropriately normal (based on pH and CO_2_), or modestly changed, which would be expected in cases where concurrent acidotic and alkalotic processes are present. Our findings are further supported by a study in critically ill human patients, with normal HH acid‐base findings, in which the SIM unmasked profound mixed metabolic effects which were neutralizing.^31^ Blood lactate was not associated with acidemic pH in PE in contrast to the findings of a previous study.^8^ The HH model also detected fewer cases with acid‐base abnormalities than did the FS approach, but more cases than the VDM did (if Δsodium and Δchloride were ignored). This might be because of the fact that the FS approach is too sensitive. Acid‐base disturbances in normal human patients have been described using a strong ion approach^32^, ^33^ and thus it could be argued that in our study, the FS approach over‐diagnosed acid‐base disease. Conversely, it seems entirely plausible, given the subtle deflections in HC$O_{3}^{-}$ seen in the face of egregious metabolic perturbations that we observed in this study that the HH model lacks sensitivity in PE where concurrent disturbances causing acidosis and alkalosis are present, as was seen in a previous study in humans.^31^

The presence of a consistent respiratory acidosis was an unexpected finding, and has not been previously described in PE. The etiology of this finding is not clear in the current study, as there was no evidence of lung pathology based on clinical evaluation of the PE puppies, and the respiratory rates were not statistically significantly different from the control group (data not shown). The authors postulated that general weakness and hypermetabolic states^34^ (because of sepsis and fever) might have contributed to the development of respiratory acidosis. Furthermore, deviations in CO_2_ were not attributable to metabolic perturbations, because they were not in the range consonant with respiratory compensation inferring a true primary disorder in this compartment. Further studies are necessary to elucidate the mechanisms involved in respiratory acidosis, in nonrespiratory/nonneurological diseases and to ascertain if any therapeutic or prognostic insights can be derived from these findings.

The preponderance of elevated UA in severe acidemia in PE was an interesting finding that warrants further investigation. Elevated UA were the most common metabolic abnormality in severe acidemia according to the both the SIM models used. Consequently, this study provides further support for the utility of SIMs in the classification of acid‐base disorders, particularly because the AG (traditionally used as part of a HH assessment) failed to demonstrate an increase in UA in our study.

The biochemical identity of the enigmatic unmeasured anions remains unclear. In addition, research in this area is scarce. One study documented an increase in certain amino acids and Kreb's cycle intermediates, in patients with elevated UA according to the SIM.^35^ In the aforementioned study, the authors demonstrated that there is a disparity between the mathematical and the analytical magnitude of UA in patients with increased UA according to the physicochemical model, suggesting that additional compounds might contribute to this poorly classified metabolic compartment.^36^ This might also be because of arithmetic overestimation, based on the calculations used, as was evident in our study, where the number of cases with UA differed according to the methodology.

A number of investigations in human medicine have documented an association between elevated UA and survival.^12^, ^13^, ^14^ This association has yet to be conclusively established in veterinary medicine. A potential association between UA and survival is intuitively logical, give that accumulation of organic acids and metabolites that are normally tightly regulated is an indication of homeostatic derangement, which suggests a systemic biochemical regulation failure. In this study, the small number of dogs with increased UA precluded survival analysis, but further studies in dogs are warranted to determine if whether UA are associated with survival in critically ill dogs.

Regarding the validity of the FS in dogs it is possible that some of the data generated by this model are inaccurate. In particular UA, which are calculated using the base excess, which is derived from the Van Slyke equation^21^, ^37^ and nomograms that were developed from studies using human blood. It was assumed that the nomograms would be the same for other species, but it has been shown that the canine nomogram differs from that of humans.^20^ It could be argued that based on this previous study the BE in other species^38^ might provide inaccurate results. While it was not within the scope of this study to validate the FS model, we felt that by deriving our cutoffs for UA according to the FS from our control dogs that we somewhat overcame this limitation, as we would have expected that these mathematical variations would have been present in both groups, but we cannot be certain of this. The differences in BE estimation because of differences in dog blood or anticoagulant used are unlikely to be of clinical relevance within the physiological pH range.^20^, ^39^ Interestingly, as could be expected, we found that when control dog data were used in the BE calculation, there were significant differences between the BE and BE_corrected_ for the PE group that was less negative than the BE (using standard formulae) group (less acidic). This is consistent with previous findings,^20^ where dog blood was shown to be slightly acidic according to the BE nomogram at pH 7.4, but our results also seem to confirm that this is unlikely to be of clinical importance. Notwithstanding this finding, we feel this study emphasizes the importance of developing and using validated methodologies and species specific reference data for comparison. In particular, the impact of protein on the BE calculation might be of significance given the species differences in plasma proteins.^38^

The authors found that by virtue of the number of variables considered, the FS provided more information and in individual cases we felt provided greater pathophysiological clarity. However, the FS was by far the most cumbersome, time consuming, and required a deeper understanding of acid‐base physiology to apply. The VDM was the easiest model to apply and understand. The HH model can be challenging to interpret in complex disorders, especially for novice clinicians as acid‐base disease can be present in the face of normal HH values. Therefore, we believe that further work is needed to assess the clinical utility of the VDM in other disease models. Future studies should also focus to ascertain if there is any therapeutic or prognostic advantage in using the SIM in dogs. Of great interest would be to determine if in the population of HH “normal” dogs identified with SIM acid‐base abnormalities, whether a SIM approach might serve as an early warning of impending homeostatic decompensation. We identified a number of limitations in our study. First, blood gases and acid‐base parameters are labile and proper sample handling is essential^17^ as variations in the type and amount of heparin used,^40^, ^41^ the presence of air bubbles^42^ improper sample mixing^43^ might affect the accuracy of the results obtained. While there is no clear gold‐standard device for blood gas analysis in veterinary medicine, bench top analyzers, that are used in reference laboratories, with daily quality controls and calibration that are used by trained laboratory personnel are likely to provide more consistent results than point‐of‐care analyzers.^44^, ^45^ Therefore, our data were collected and processed by a single experienced operator, using a bench top blood gas analyzer. A second limitation was the relatively small control dog size for data comparison. Establishment of a reference interval for this study with confidence levels would have been ideal. Reference intervals have already been established for dogs^19^, ^46^ and so the purpose of our control group was to ensure that the healthy dogs were within the limits of normal published values, and that our control dog data did not differ significantly from published ranges.

## 5 CONCLUSIONS

In conclusion our study showed that the acid‐base changes of PE are multifactorial and easier to explain using the SIM. Furthermore we showed that the AG is unreliable in detecting UA in dogs in PE and likely lacks sensitivity. Lastly, our study demonstrated that much more research in the area of acid‐base medicine is needed, and also greater clarity is needed within the reference texts available to veterinarians to avoid confusion when it comes to the dazzling array of equations and analyses available because most clinicians do not have the mathematical acumen to grasp the difference between the calculations available for variables such as UA.

## CONFLICT OF INTEREST DECLARATION

Authors declare no conflict of interest.

## OFF‐LABEL ANTIMICROBIAL DECLARATION

Authors declare no off‐label use of antimicrobials.

## INSTITUTIONAL ANIMAL CARE AND USE COMMITTEE (IACUC) OR OTHER APPROVAL DECLARATION

Authors declare no IACUC or other approval was needed.

## HUMAN ETHICS APPROVAL DECLARATION

Authors declare human ethics approval was not needed for this study.
